# Validation of a Questionnaire to Assess the Usability of and User Experience with Mobile Health Applications

**DOI:** 10.3390/healthcare12232328

**Published:** 2024-11-21

**Authors:** Anna de Dios López, Jordi Real, Claudia Meza, Alicia Borras-Santos, Roberto Collado-Borrell, Vicente Escudero-Vilaplana, Mar Gomis-Pastor

**Affiliations:** 1 Digital Health Validation Center, Hospital de la Santa Creu i Sant Pau, Sant Pau Campus Salut Barcelona, 08001 Barcelona, Spain; adedios@santpau.cat (A.d.D.L.); jrealg@santpau.cat (J.R.); aborrass@santpau.cat (A.B.-S.); 2 Institut de Recerca Sant Pau (IR SANT PAU), Sant Quintí 77 79, 08041 Barcelona, Spain; 3 Pharmacy Department, Hospital de la Santa Creu i Sant Pau, IIB Sant Pau, 08001 Barcelona, Spain; 4 Department of Medicine, Universitat Autònoma de Barcelona, 08193 Barcelona, Spain; claudia.mezabcn@gmail.com; 5 Stroke Research, Vall d’Hebron Institut de Receca, 08035 Barcelona, Spain; 6 Pharmacy Department, Hospital General Universitario Gregorio Marañón, 28007 Madrid, Spain; roberto.collado@salud.madrid.org (R.C.-B.); vicente.escudero@salud.madrid.org (V.E.-V.)

**Keywords:** user-centred design, user experience, mobile health, mHealth, healthcare application, comprehensive healthcare, surveys and questionnaires, patient satisfaction

## Abstract

Background/objectives: The growing use of mobile health (mHealth) applications needs reliable tools to assess their usability and user experience in clinical practice to improve the digital health (eHealth) interventions and ensure engagement, as higher engagement is often linked to increased efficacy of healthcare interventions. This study aimed to validate the patient Satisfaction and Usability with APPs questionnaire (pSUAPP), a multidimensional tool designed for the comprehensive assessment of mHealth applications, particularly for the integrated follow-up of patients with chronic diseases. Methods: A validation study was conducted between August and December 2022 with 85 participants from two hospitals in Spain, who completed the pSUAPP questionnaire, comprising 27 Likert-like items across four dimensions: first contact, registration, features and overall experience, and 1 open question. The questionnaire was validated by a panel of 11 experts and further assessed for psychometric properties. Results: The mean pSUAPP score was 79.0 (SD = 12.0), indicating high usability and positive user experience, with the highest scores in the ‘features’ dimension. The pSUAPP demonstrated moderate correlation with the System Usability Scale (SUS) and high reliability (Cronbach’s alpha and omega t > 0.9). A reproducibility analysis showed negligible changes between repeated measures. Conclusions: The pSUAPP questionnaire was found to be a robust tool for evaluating mHealth app usability and user experience, with potential application across various clinical settings.

## 1. Introduction

Digital health technology refers to a broad range of technologies and tools that use digital solutions to enhance healthcare delivery, improve the healthcare workflow, and boost personalized patient care [1,2]. In this context, the use of mobile health (mHealth) represents a way of integrating digital health technology into the healthcare sector through mobile devices such as smartphones or tablets [3].

The use of digital health technologies has grown in the past years, especially during the COVID-19 pandemic, with the development of over 300,000 mHealth applications for the management of different conditions [4,5]. However, different problems and barriers related to their use, such as usability, privacy, security, cost, device compatibility, among others, might arise [6]. Among these, usability, defined as the extent to which a system can be used effectively, efficiently, and satisfactorily by specific users in a given context, is crucial for ensuring the engagement and adoption of an mHealth app, and ultimately, this impacts the empowerment of individuals with their treatment and disease, as well as the effectiveness of eHealth interventions provided by healthcare professionals through mHealth platforms [6,7].

Currently, despite the importance of ensuring the usability of and patient experience with mHealth apps, there are few validated tools for use in clinical practice [8]. The System Usability Scale (SUS), one of the earliest tools developed to measure usability, remains widely used, although it is a generic questionnaire, and it is not tailored to health nor digital technologies [9]. Other tools have been specifically designed to assess the usability of mHealth. For instance, Zhou et al. developed and validated the mHealth app usability questionnaire (MAUQ) based on other usability questionnaires used in health and non-health settings [10]. Similarly, Schnall et al. validated the Health IT Usability Evaluation Scale (Health-ITUES) for the assessment of the usability of mHealth technologies, initially designed for HIV patients [11]. Additionally, a recent study identified that the Health-ITUES and the Measurement Scales for Perceived Usefulness and Perceived Ease of Use (MSPUPE) have been well validated and primarily emphasize usability. Conversely, they suggest that the Mobile App Rating Scale (MARS) covers a broader range of quality dimensions and has been validated across numerous mHealth applications, leading to its widespread use for mHealth evaluations [12]. To further enhance mHealth usability assessments, recent research has focused on the development of comprehensive user experience evaluation scales. For instance, Kim et al. (2024) validated a scale based on five factors that proved effective for evaluating user experience in mHealth apps and has allowed for continuous improvements in their design, ensuring that these applications meet patient needs and provide comprehensive user feedback [13]. In another recent study, the authors proposed a usability evaluation model for mHealth apps that integrates both subjective and objective metrics, recommending a risk-based approach for optimizing interaction and information access, while also addressing key usability risks [14]. However, these questionnaires have constraints, including their limited adaptability to diverse clinical workflows, potential lack of sensitivity to the specific needs of various patient populations [10,12], and the possibility that they may not fully capture the complexity and variability of real-world healthcare environments [11,12]. Additionally, these tools may require significant customization to be effectively implemented in different medical specialties, which can be time-consuming and resource-intensive [9].

In response to the specific need for a tool to assess the usability, satisfaction, and overall user experience with an mHealth app focused on the comprehensive clinical follow-up of chronic patients with a complex therapeutic management and diverse care needs, a multidimensional patient-reported questionnaire was previously developed [15]. This first empiric framework laid the foundation for the design and validation of the patient Satisfaction and Usability with APPs (pSUAPP) questionnaire. The primary aim of this study was to validate this novel questionnaire as a tool to assess the usability of and the satisfaction and overall patient experience with an mHealth application designed for the remote and integral clinical management of complex chronic patients.

## 2. Methods

### 2.1. Study Design

This is a validation study, involving participants from several eHealth studies focused on different pathologies. The secondary objectives of this study were to assess to the usability of and overall user experience with an mHealth application and to evaluate the psychometric properties of a newly developed self-administered questionnaire to assess the usability of and overall user experience with an mHealth application. The eHealth studies included the following: MY-Medula (Ethics Committee number IIBSP-EME-2019-44), eMig (IIBSP-MIG-2020-09), On-Communities (PR343/18), ePGx (IIBSP-EPG-2020-84), mHeart (IIBSP-MHE-2014-55), and mTAR (IIBSP-ARV-2020-78).

All the studies were conducted in accordance with the Declaration of Helsinki [16] and Good Clinical Practice guidelines [17].

### 2.2. Questionnaire

The pSUAPP questionnaire (Supplementary Materials Table S1) consists of 27 Likert-like items, grouped into the following dimensions: first contact (usability and first contact with the health application; 6 items), registration (data entry in the health application; 8 items), features (health application functionalities; 6 items) and overall experience (user experience with the health application; 7 items), and 1 open-ended question (nº 21). The questionnaire is included in its translated version corresponding to the Spanish version (Supplementary Materials Table S1).

The process to design and validate the questionnaire was as follows: (1) a theoretical background was established through a review of the literature; (2) the questionnaire was created with the help of an expert panel of 11 professionals following the Delphi methodology; (3) pilot testing of the questionnaire was performed; (4) improvements were implemented in the questionnaire given the received responses; and (5) the final version of the questionnaire was sent to the participants and the statistical analysis was performed including the factorial analysis, dimensionality, reproducibility, and reliability.

The questionnaire was initially designed by three clinical experts following the COSMIN [18] and CHERRIES [19] guidelines and subsequently validated by a panel of 11 experts from different specialties given their clinical experience in the use of telemedicine, following a Delphi methodology with two rounds to reach consensus. To ensure a heterogeneous perspective, the expert panel included rheumatology physicians (*n* = 3), hospital pharmacists (*n* = 3), transplant specialists nurses (*n* = 2), haematology physicians (*n* = 2), and a biomedical engineer (*n* = 1). The experts evaluated each question, indicating, among other aspects, if there were useful, intuitive, short, and easy to answer, with adaptive language, or whether the questions were understandable. The professionals selected for the validation panel were required to have at least a masters’s or doctoral degree, or a minimum of five years of experience in the use of mHealth technologies. Additionally, they needed clinical experience, expertise in scientific research, and teaching. The methodology recommended by the COSMIN Study Design checklist [20] was used to evaluate the pSUAPP questionnaire by assessing the psychometric properties of the app, the convergent/divergent validity and its reliability, reproducibility, and dimensionality. CHERRIES Checklist guidelines were also used as a validated tool to ensure the quality of the questionnaire [19,21] (Supplementary Materials Table S2).

### 2.3. Participants

This study included patients from the Haematology, Oncology, Cardiology, Neurology, and Internal Medicine departments of two high-complexity tertiary care hospitals: Hospital de la Santa Creu i Sant Pau in Barcelona and Hospital General Universitario Gregorio Marañón in Madrid. The study was conducted between August and December 2022.

The questionnaire was distributed among participants from both hospitals that had previously used the platform and who had participated in the following studies: MY-Medula, eMig, On-Communities, ePGx, mHeart, and mTAR (*n* = 106). After excluding 21 incomplete responses, the final analysable sample comprised 85 participants. Eligible participants were adult patients with a complex chronic condition who had prior experience using an mHealth application and were capable of independently managing the app for remote and comprehensive clinical monitoring of their health condition. Patients were invited to participate in the study by their healthcare providers, including pharmacists, nurses, and doctors. The invitation was sent by these professionals through a messaging system that arrived directly to the patient’s mobile application. Written informed consent was obtained from all participants prior to completing the questionnaire. The survey was designed in Spanish and conducted by patients electronically via mHealth applications already in use by the participants.

Patients used different versions of the same app developed by the technology provider TrilemaSalud SL. These versions were adaptations of the technology to different chronic pathologies and patient pathways. All versions offered comprehensive monitoring of patients’ chronic diseases and comorbidities, including communication, lifestyle monitoring, and pharmacological treatments. TrilemaSalud SL is a Spanish company that has experience in the world of Health and Education, offering different types of digital solutions that are used by several national and international hospitals. It monitors the effectiveness of training and education on patient outcomes, helping institutions assess the impact of their programmes and improve healthcare delivery. Some of the key features are outcome tracking and reporting, patient feedback integration, and data-driven decision support [22].

### 2.4. Variables

The main variable of this study was the pSUAPP score, an overall summary measure ranging from 0 to 100, where higher scores indicate better outcomes. The pSUAPP score was calculated by assigning individual scores to each item as follows: for positive items [1,2,3,4,5,6,7,8,9,10,11,12,13,14,15,16,17,18,19,20,21,22,23,24], 1 was subtracted from the user response, which was in a scale from 1 to 5, and therefore, the results were from 0 to 4; for negative items [25,26], the user responses were subtracted from 5, which then would be translated into an inverse scale from 0 to 4. Thus, all responses were scaled to 0–4, with 0 representing the most negative response and 4 the most positive. The overall pSUAPP score was then calculated by summing of the converted responses for each user, multiplying by 2.5, and ultimately obtaining the range of possible values from 0 to 100. Each dimension (first contact, registration, features, and experience) was computed using the same scoring method.

Handling missing data: out of the 27 scale items, 15 were deemed mandatory [1,2,3,4,5,6,13,18,21,22,23,24,25,26,27], suitable to all mHealth application. If any of these items were incomplete, the overall score could not be calculated and was marked as missing. Conversely, unanswered optional items were excluded if they had missing data, allowing the calculation of the overall score in these cases. Additionally, a subset version called pSUAPP-reduced was created using 6 essential items [4,13,18,21,22,27].

For reproducibility assessment, a test-retest was carried out, in which participants were asked to complete the questionnaire twice, with both completions separated by approximately 10 days.

Secondary variables included participants’ sociodemographic characteristics (age, sex, education) and the System Usability Scale (SUS) score, calculated as previously described [23].

### 2.5. Statistical Analysis

Mean, standard deviation (SD), frequency, and percentages were used to describe the sociodemographic characteristics, while mean, SD, median, minimum, and maximum were used to describe the pSUAPP and SUS scores.

The visualization of responses by domain was represented by a Diverging Stacked Bar Chart [24].

The convergent validity analysis evaluated the correspondence between the pSUAPP scale and its subdimensions with the SUS by estimating the intraclass correlation coefficient (ICC) and the 95% confidence interval (95% CI). Differences between both scales and the standardized difference were evaluated with a paired Student’s *t*-test. Potential differences in global pSUAPP between sociodemographic groups were analysed with Student’s *t* or ANOVA test.

Reliability was assessed evaluating the internal consistency of the questionnaire using Cronbach’s alfa and omega coefficients. Omega can be dived into omega *h* (model-based hierarchical estimate of the general factor saturation of a scale) and omega *t* (model-based estimate of the total reliability of a scale) [25]. This analysis was performed globally and in specific sociodemographic groups.

To assess pSUAPP’s reproducibility, the differences between the first and the second time participants completed the questionnaire were evaluated using the mean estimate of the change and 95% CI, the standardized difference (Cohen’s effect), and the ICC. The confidence level for the correlation and difference with respect to the SUS was estimated using the cor.test and t.test functions from the R package, both of which utilize Student’s t-distribution for their estimations.

To analyse the dimensionality (structural validity), the correlation between domains and overall score was established, and a factor analysis was performed with the responses to the items. The exploratory factor analysis was performed with the fa() function from the psych package to investigate the latent structure of variables and reduce data dimensionality. Four factors were specified, with the weighted least squares (WLS) method used to minimize residuals between observed and reconstructed correlation matrices. To enhance interpretability, we applied a varimax rotation, an orthogonal method that clarifies factor structures by maximizing the variance of squared loadings, resulting in a clearer factor pattern where variables load primarily on one factor. Factor loadings exceeding 0.3 were considered significant for interpretation. The analysis outputs include factor loadings for each variable, the proportion of variance explained, and model fit indices (such as the root mean square of the residuals, RMSR), offering insights into the dataset’s underlying dimensions (Table S3).

The statistical analyses were carried out using the R package version 4. All *p*-values < 0.05 were considered statistically significant.

## 3. Results

### 3.1. Participants Characteristics

A total of 85 participants completed the pSUAPP questionnaire, with a mean age of 52.0 years (SD = 11.5). All the participants were patients of oncology (mama and colorectal cancer), haematology (myeloma and bone marrow), neurology (migraine), cardiology (heart transplant), and internal medicine (VIH). Most of them were women (56.5%) and had higher education (62.4%) (Table 1).

### 3.2. pSUAPP Psychometric Evaluation

**Figure 1:**
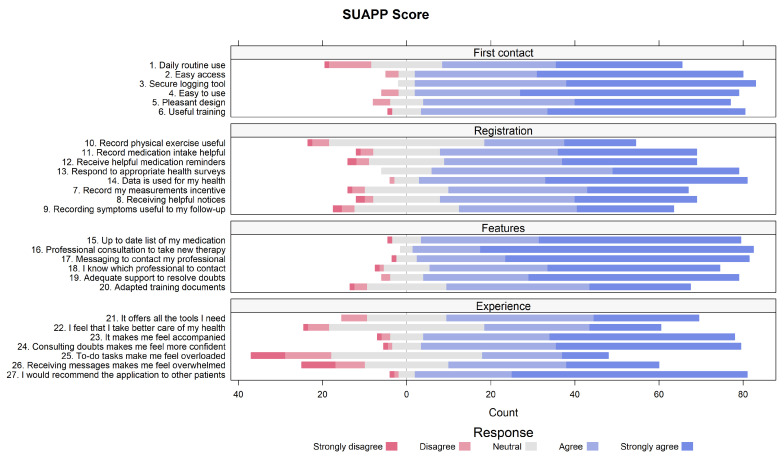
Diverging stacked bar chart of the pSUAPP scores obtained in the different dimensions.

The mean (SD) overall pSUAPP score obtained was 79.0 (12.0), which was statistically significantly higher than the mean (SD) SUS score (70.4 [13.4]; *p* < 0.001), reported by the same participants (Table 2). Among the pSUAPP dimensions, the “features” domain received the highest score (Table 2 and Figure 1).

When the pSUAPP score was analysed according to the participants’ sociodemographic characteristics, no statistically significant differences were observed (Supplementary Materials Table S4).

### 3.3. Convergent Validity

The correlation analysis between pSUAPP and SUS scores revealed a statistically significant correlation between the SUS score and both the overall pSUAPP score and the scores of individual pSUAPP domains, with the highest correlation observed in the “experience” domain (Table 3).

Additionally, the mean difference between the SUS score and both the overall pSUAPP score and the scores of individual pSUAPP domains were statistically significant in all cases (Table 3).

### 3.4. Reliability

Cronbach’s alfa and omega *t* coefficients indicated high reliability, with values over 0.9 for both overall and for each sociodemographic subgroup; no major differences in reliability measures were observed between groups. Omega h showed lower values in all cases (Table 4).

### 3.5. Reproducibility

A total of 40 participants completed the pSUAPP questionnaire a second time, with a median interval of 10 days between administrations.

The mean change (95% CI) in the overall pSUAPP score showed a reduction of 0.59 (−1.65, 2.84) units, with a Cohen’s effect size of 0.05. All standardized differences were considered negligible (|d| < 0.2), and overall, correlations were high (ICC > 0.7) (Table 5).

### 3.6. Dimensionality

**Figure 2:**
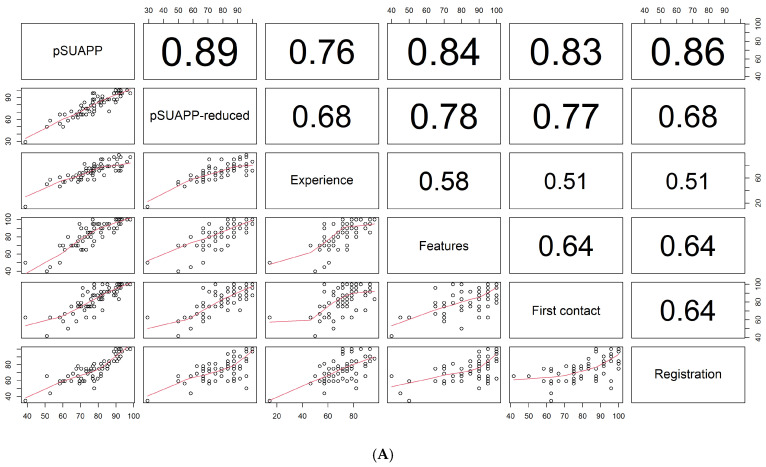

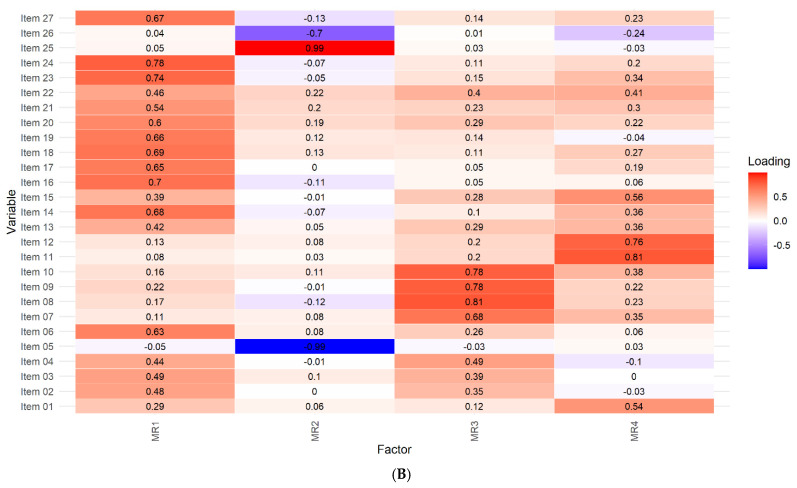
Correlation analysis between domains and the overall score (**A**) and factor analysis (**B**).

The analysis of the correlation between domains and the overall score showed that the domain “registration” had the highest correlation with the overall score. The domain “experience” showed the lowest correlation with the other domains (r < 0.60) (Figure 2A).

The factor analysis showed that the first three factors explain 82% of the variability. In the first factor, the items with the highest weight were those related to the “features” and “experience” domains, while in the third factor, they were those related to the “registration” domain (Figure 2B).

## 4. Discussion

The growing use of mHealth applications for chronic complex patients highlights the need for validated tools to assess usability and patient experience. Usability is key to the success of these technologies, impacting patient engagement and health outcomes. This study addresses this need by validating the pSUAPP questionnaire, designed specifically for evaluating these aspects in mHealth applications.

Several established frameworks have been proposed for the design and evaluation of digital health interventions, such as Health Information Technologies, eHealth, and mHealth [24,27,28]. These frameworks provide valuable guidelines for assessing health information technologies, including mHealth applications. Our findings on patient experience and usability align with the recommendations outlined in these frameworks, emphasizing the importance of user-centred design in mHealth applications.

The results of our study indicate that the pSUAPP questionnaire is an effective tool for assessing mHealth usability with an overall mean score of 79.0, indicating a generally positive experience. The “features” dimension received the highest score, suggesting that users particularly valued the apps’ functionalities. No statistically significant differences between sociodemographic groups were observed, which may indicate that the questionnaire is measuring the construct consistently across different groups showing the tool’s broad applicability. In addition, the pSUAPP scale exhibited a moderate correlation with the SUS questionnaire, high reliability coefficients (Cronbach’s alpha > 0.9), and good replicability metrics (Cohen’s effect size 0.05).

Different studies indicate that mHealth applications with good usability could help patients understand and use them more effectively and easily, ultimately leading to a better engagement [29,30]. Despite the increasing efforts to facilitate ways to measure engagement in certain healthcare areas such as mental health [12,31], there is still work to do in this context, as only a few questionnaires provide the evaluation of engagement in different pathologies [32].

Despite the availability of newer tools, the SUS remains the most frequently utilized questionnaire, followed by the mobile application rating scale (MARS) [12,33]. These tools, like the pSUAPP, employ a 5-point Likert scale and have showed a high level of reliability (SUS: alpha of 0.91; MARS: omega 0.79–0.93) [9,34]. In addition, the correlation results confirmed the low specificity of the SUS questionnaire in comparison to pSUAPP. Although not the most frequently used, the MAUQ, specifically developed for interactive mHealth applications, also showed a high level of reliability, with a Cronbach’s alpha value of 0.93 [10]. In this context, the results of our study showed that the pSUAPP questionnaire is an alternative to previously validated questionnaires.

Regarding some limitations of our study, first, the survey was conducted on applications created by the same technology developer, which may constrain the generalizability of the obtained results. Nevertheless, the patients included had a wide variety of pathologies, were being followed up in two centres, the Hospital de la Santa Creu i Sant Pau in Barcelona and Hospital General Universitario Gregorio Marañón in Madrid, and were using different versions of the app adapted to the different care routes and pathologies. strengthening the validation of the findings. In addition, we also tested different applications from the same developer in different populations, which counteracted this limitation. Second, the questionnaire included 27 items (plus 1 open question), which could result in needing a long time to complete it and could ultimately affect the response rate and data quality. However, having a high number of items can provide a more comprehensive coverage, contributing to the validity and reliability of the questionnaire. Alternatively, a reduced and more manageable version of pSUAPP comprising six items, called pSUAPP reduced, was also proposed to overcome this limitation. Third, the order of the items and the limited use of reverse-scored questions included in the questionnaire could introduce response bias. However, the inclusion of reverse questions could help to detect potential response bias and improve the validity of the results by providing a more balanced perspective on the items assessed. Fourth, the sample did not include senior citizens. This fact is due to the inclusion criteria that selected patients with prior experience using mHealth applications. Even though age is not a factor for excluding patients in our centres, this population is typically underrepresented when technology is involved. Finally, although it is considered that the sample was sufficient for the purpose of this study, a larger sample of patients and responses in the second round would have benefited the accuracy of the results.

The pSUAPP questionnaire shows strong potential for expansion in multiple directions. On the one hand, it could be adapted for integration into mHealth applications developed by other technology providers, broadening its reach and applicability. Additionally, the versatility of pSUAPP makes it suitable for its use in a wide range of pathologies, enhancing its relevance across different medical areas. Translating the pSUAPP to English and other languages could also facilitate its global adoption, enabling the evaluation of the user experience and satisfaction with health apps worldwide.

In conclusion, the pSUAPP questionnaire represents an effective tool for assessing the usability of and user experience with mHealth applications, especially for the integrated management of patients with chronic diseases. The specificity and applicability of the questionnaire to different pathologies is also a key strength of the pSUAPP questionnaire in comparison to other previously validated tools. It makes the mHealth application include specific variables for each pathology, increasing its specificity compared to other tools. The use in different settings of this new tool to measure patient satisfaction and experience with health apps, and the expansion of this study to include wider mobile health approaches could provide further insights into its applicability and potential use across various clinical settings.

## 5. Conclusions

The pSUAPP questionnaire represents an effective tool for assessing the usability of and user experience with mHealth applications, especially for the integrated management of patients with chronic diseases. The specificity and applicability of the questionnaire to different pathologies is also a key strength of the pSUAPP questionnaire in comparison to other previously validated tools. It makes the mHealth application to include specific variables for each pathology, increasing its specificity compared to other tools. The use of this new tool in different settings to measure patient satisfaction and experience with health apps, and the expansion of this study to include wider mobile health approaches, could provide further insights into its applicability and potential use across various clinical settings.

## Figures and Tables

**Table 1:** Characteristics of the study population.

	Participants (*n* = 85)	*n* Valid
Age (years), mean (SD)	52.0 (11.5)	
Age (years), median (min, max)	53.0 (19.6, 75.1)	83
Age groups (years), *n* (%)		83
18–44	23 (27.7)	
45–64	51 (61.4)	
>65	9 (10.8)	
Sex, *n* (%)		85
Men	37 (43.5)	
Women	48 (56.5)	
Education, *n* (%)		85
Higher education	53 (62.4)	
Secondary school	20 (23.5)	
Primary school	12 (14.1)	

SD: standard deviation.

**Table 2:** Overall and individual domain mean and median SUAPP scores.

	Score		Missing, *n* (%)
	Mean (SD)	Median (Min, Max)	
pSUAPP	79.0 (12.0)	77.8 (38.9, 100.0)	-
pSUAPP-reduced	78.0 (13.5)	80.0 (25.0, 100.0)	-
Domains			
First contact	83.2 (14.1)	87.5 (41.7, 100.0)	-
Registration	75.3 (15.7)	75.0 (34.4, 100.0)	13 (15.3)
Features	85.2 (14.1)	90.0 (40.0, 100.0)	7 (8.2)
Experience	73.4 (13.5)	75.0 (14.3, 100.0)	-

**Table 3:** Correlation and difference between SUS score and overall and individual domain pSUAPP scores.

	Correlation vs. SUS		Difference vs. SUS	
	Pearson’s Coefficient (95% CI)	*p*-Value	Mean (95% CI)	*p*-Value
pSUAPP	0.552 (0.384, 0.685)	<0.001	−8.62 (−11.23, −6.01)	<0.001
pSUAPP-reduced	0.533 (0.361, 0.670)	<0.001	−10.79 (−13.69, −7.90)	<0.001
First contact	0.555 (0.388, 0.687)	<0.001	−12.85 (−15.66, −10.05)	<0.001
Registration	0.318 (0.093, 0.512)	<0.001	−5.58 (−9.63, −1.53)	0.008
Features	0.378 (0.169, 0.554)	<0.001	−15.42 (−18.88, −11.95)	<0.001
Experience	0.604 (0.449, 0.724)	<0.001	−3.02 (−5.60, −0.44)	0.022

CI: confidence interval.

**Table 4:** Reliability measures in the overall study sample and according to participants sociodemographic characteristics.

	Alpha	Omega *t*	Omega *h*	*n*
Overall	0.93	0.94	0.63	85
Age groups (years)				83
18–44	0.94	0.95	0.41	23
45–64	0.94	0.95	0.65	51
>65	0.92	0.96	0.14	9
Sex				85
Men	0.95	0.96	0.67	37
Women	0.92	0.94	0.45	48
Education				85
Higher education	0.94	0.95	0.54	53
Secondary school	0.90	0.93	0.25	20
Primary school	0.95	0.96	0.65	12
Subscale				
pSUAPP reduced	0.81	0.84	0.63	85
Experience	0.79	0.88	0.11	85
Features	0.84	0.87	0.67	78
First contact	0.81	0.84	0.63	85
Registration	0.87	0.91	0.56	72

Omega *h*: the model-based hierarchical estimate of the general factor saturation of a scale; Alpha: the conventional alpha statistic (which is not model-based); Omega *t*: a model-based estimate of the total reliability of a scale.

**Table 5:** Comparison between first and second time overall and individual domain pSUAPP scores and correlation.

	Pre	Post	Change (95% CI)	*p*-Value	Cohen’s Effect Size	ICC
pSUAPP	79.96	79.37	0.59 (−1.65, 2.84)	0.60	0.05	0.81
pSUAPP-reduced	80.83	80.00	0.83 (−2.10, 3.76)	0.57	0.06	0.75
First contact	83.96	80.52	3.44 (−0.31, 7.18)	0.07	0.23	0.68
Registration	74.51	79.10	−1.62 (−5.29, 2.05)	0.37	−0.12	0.78
Features	84.58	83.38	0.42 (−2.70, 3.53)	0.79	0.03	0.73
Experience	76.34	76.96	−0.63 (−3.70, 2.45)	0.68	−0.05	0.69

CI: confidence interval; ICC: intraclass correlation coefficient.

## Data Availability

The data used to support this study are available from the corresponding author upon request.

## Supplementary Materials

The following supporting information can be downloaded at: https://www.mdpi.com/article/10.3390/healthcare12232328/s1, Table S1: Patient questionnaire on Satisfaction with the Use of Health Applications (pSUAPP); Table S2: Checklist for Reporting Results of Internet E-Surveys (CHERRIES) [19]. Table S3: Summary model factor analysis; Table S4: SUS score and overall and individual domain mean pSUAPP scores according to participants sociodemographic characteristics.

## References

[1] WHO (2019). WHO Guideline: Recommendations on Digital Interventions for Health System Strengthening.

[2] Sharma A., Harrington R.A., McClellan M.B., Turakhia M.P., Eapen Z.J., Steinhubl S., Mault J.R., Majmudar M.D., Roessig L., Chandross K.J. (2018). Using Digital Health Technology to Better Generate Evidence and Deliver Evidence-Based Care. J. Am. Coll. Cardiol..

[3] Bradway M., Carrion C., Vallespin B., Saadatfard O., Puigdomènech E., Espallargues M., Kotzeva A. (2017). mHealth Assessment: Conceptualization of a Global Framework. JMIR Mhealth Uhealth.

[4] Iribarren S.J., Akande T.O., Kamp K.J., Barry D., Kader Y.G., Suelzer E. (2021). Effectiveness of Mobile Apps to Promote Health and Manage Disease: Systematic Review and Meta-analysis of Randomized Controlled Trials. JMIR Mhealth Uhealth.

[5] O’Connor M., Bowles K.H. (2021). Telehealth and mHealth. Res. Nurs. Health.

[6] Giebel G.D., Speckemeier C., Abels C., Plescher F., Börchers K., Wasem J., Blase N., Neusser S. (2023). Problems and Barriers Related to the Use of Digital Health Applications: Scoping Review. J. Med. Internet Res..

[7] Liew M.S., Zhang J., See J., Ong Y.L. (2019). Usability Challenges for Health and Wellness Mobile Apps: Mixed-Methods Study Among mHealth Experts and Consumers. JMIR Mhealth Uhealth.

[8] Giebel G.D., Speckemeier C., Schrader N.F., Abels C., Plescher F., Hillerich V., Wiedemann D., Börchers K., Wasem J., Blase N. (2024). Quality assessment of mHealth apps: A scoping review. Front. Health Serv..

[9] Brooke J. (1996). SUS: A quick and dirty usability scale. Usability Eval. Ind..

[10] Zhou L., Bao J., Setiawan I.M.A., Saptono A., Parmanto B. (2019). The mHealth App Usability Questionnaire (MAUQ): Development and Validation Study. JMIR Mhealth Uhealth.

[11] Schnall R., Cho H., Liu J. (2018). Health Information Technology Usability Evaluation Scale (Health-ITUES) for Usability Assessment of Mobile Health Technology: Validation Study. JMIR Mhealth Uhealth.

[12] Muro-Culebras A., Escriche-Escuder A., Martin-Martin J., Roldán-Jiménez C., De-Torres I., Ruiz-Muñoz M., Gonzalez-Sanchez M., Mayoral-Cleries F., Biró A., Tang W. (2021). Tools for Evaluating the Content, Efficacy, and Usability of Mobile Health Apps According to the Consensus-Based Standards for the Selection of Health Measurement Instruments: Systematic Review. JMIR Mhealth Uhealth.

[13] Kim G., Hwang D., Park J., Kim H.K., Hwang E.S. (2024). How to Design and Evaluate mHealth Apps? A Case Study of a Mobile Personal Health Record App. Electronics.

[14] Shen Y., Wang S., Shen Y., Tan S., Dong Y., Qin W., Zhuang Y. (2024). Evaluating the Usability of mHealth Apps: An Evaluation Model Based on Task Analysis Methods and Eye Movement Data. Healthcare.

[15] Gomis-Pastor M., Roig E., Mirabet S., J T.D.P., Conejo I., Feliu A., Brossa V., Lopez L., Ferrero-Gregori A., Barata A. (2020). A Mobile App (mHeart) to Detect Medication Nonadherence in the Heart Transplant Population: Validation Study. JMIR Mhealth Uhealth.

[16] World Medical Association (2024). Wma Declaration of Helsinki—Ethical Principles for Medical Research Involving Human Participants. https://www.wma.net/policies-post/wma-declaration-of-helsinki/.

[17] European Medicines Agency (EMA) (2016). Guideline for Good Clinical Practice. https://www.ema.europa.eu/en/documents/scientific-guideline/ich-guideline-good-clinical-practice-e6r2-4-step-2b_en.pdf.

[18] Mokkink L.B., Prinsen C., Patrick D.L., Alonso J., Bouter L., De Vet H.C., Terwee C.B., Mokkink L. (2018). COSMIN methodology for systematic reviews of Patient-Reported Outcome Measures (PROMs). User Man..

[19] Eysenbach G. (2004). Improving the Quality of Web Surveys: The Checklist for Reporting Results of Internet E-Surveys (CHERRIES). J. Med. Internet Res..

[20] Mokkink L.B., Prinsen C.A.C., Patrick D.L., Alonso J., Bouter L.M., de Vet H.C.W., Terwee C.B. (2019). COSMIN Study Design Checklist for Patient-Reported Outcome Measurement Instruments.

[21] Lopez-Rodriguez J.A. (2019). Improving the quality of Spanish web surveys: Spanish adaptation of the checklist for reporting results of internet e-surveys (CHERRIES) to the Spanish context. Atención Primaria.

[22] TrilemaSalud. https://www.trilemasalud.com/.

[23] Ghazarian A. Measuring Usability with System Usability Scale (SUS). https://webdesignviews.com/measuring-usability-with-system-usability-scale-sus/.

[24] Christopoulou S.C., Kotsilieris T., Anagnostopoulos I. (2018). Assessment of Health Information Technology Interventions in Evidence-Based Medicine: A Systematic Review by Adopting a Methodological Evaluation Framework. Healthcare.

[25] Robbins N., Heiberger R. Plotting Likert and Other Rating Scales. Proceedings of the 2011 Joint Statistical Meeting.

[26] Revelle W., Condon D.M. (2019). Reliability from α to ω: A tutorial. Psychol. Assess.

[27] Ogundipe A., Sim T.F., Emmerton L. (2023). Health information communication technology evaluation frameworks for pharmacist prescribing: A systematic scoping review. Res. Soc. Adm. Pharm..

[28] Ogundipe A., Sim T.F., Emmerton L. (2023). Development of an evaluation framework for health information communication technology in contemporary pharmacy practice. Explor. Res. Clin. Soc. Pharm..

[29] Anders C., Moorthy P., Svensson L., Muller J., Heinze O., Knaup P., Wallwiener M., Deutsch T.M., Le T.V., Weinert L. (2024). Usability and User Experience of an mHealth App for Therapy Support of Patients With Breast Cancer: Mixed Methods Study Using Eye Tracking. JMIR Hum. Factors.

[30] Zapata B.C., Fernandez-Aleman J.L., Idri A., Toval A. (2015). Empirical studies on usability of mHealth apps: A systematic literature review. J. Med. Syst..

[31] Kopka M., Camacho E., Kwon S., Torous J. (2023). Exploring how informed mental health app selection may impact user engagement and satisfaction. PLoS Digit. Health.

[32] Rana R., Ibrahim B.B., Huri H.B.Z., Wahab I.B.A., Govindaraju K., Shukeri M.S.M., Ng C.K., Ong S.C. (2024). Development and validation of the mobile adherence satisfaction scale (MASS) for medication adherence apps. Res. Soc. Adm. Pharm..

[33] Hajesmaeel-Gohari S., Khordastan F., Fatehi F., Samzadeh H., Bahaadinbeigy K. (2022). The most used questionnaires for evaluating satisfaction, usability, acceptance, and quality outcomes of mobile health. BMC Med. Inform. Decis. Mak..

[34] Terhorst Y., Philippi P., Sander L.B., Schultchen D., Paganini S., Bardus M., Santo K., Knitza J., Machado G.C., Schoeppe S. (2020). Validation of the Mobile Application Rating Scale (MARS). PLoS ONE.

