# Supplementary material for: Validation of a Questionnaire to Assess the Usability of and User Experience with Mobile Health Applications
Source: Healthcare (Basel). 2024 Nov 21;12(23):2328. doi: 10.3390/healthcare12232328 (PMC11640890; doi:10.3390/healthcare12232328)
Supplement: Supplementary file 1 [file healthcare-12-02328-s001.zip › healthcare-3272297-supplementary.pdf]

healthcare

MDPI

Table S1. Patient questionnaire on Satisfaction with the Use of ealth Applications (pSUAPP)

Below, we present 28 statements that we ask you to read and answer carefully. We invite you to select the option that best represents your opinion about each statement: strongly agree, agree, neither agree nor disagree, disagree, strongly disagree. In addition, you will find the option "does not apply to me". You can select it when you consider that the health application you are using does not have the functionality referred to in the statement.

	Strongly agree	Agree	Neither agree nor disag ree	Disagree	Strongly disag ree	Not applica ble to my case
USABILITY AND FIRST CONTACT WITH THE HEALTH APPLICATION						
1. Accessing the application is part of my daily routine.						
2. Accessing the application is easy for me						
3. I find the application a secure tool to record my health data.						
4. I find the application easy to use						
5. I find the design of the application pleasant						
6. The initial training I received on how to use the application was helpful.						
DATA ENTRY IN THE HEALTH APPLICATION						
7. Recording my measurements (weight, blood pressure, etc.) encourages me to keep better track of my measurements.						
8. Receiving prompts to remind me to record my vitals is helpful.						
9. Recording symptoms that occur to me (headache, diarrhea, etc.) is useful for my follow-up.						
10. Recording the physical exercise I do is useful to me.						
11. I find it helpful to record my medication intake.						
12. I find it helpful to receive reminders to take my medication.						
13. Responding to health surveys through the application is adequate for me.						
14. I consider that the data I register is used by my health care team to better monitor my health.						
HEALTH APPLICATION FUNCTIONALITIES						
15. I find it useful to have an updated list of my medications.						
16. I find it useful to consult with my professional if I can take a new therapy (medication, plants, infusions, homeopathy or other alternative therapy).						
17. I find messaging useful for contacting my health care professional.						
18. I know which practitioner I should contact at any given time to resolve my doubts.						

19. The support I have received to resolve doubts and incidents with the use of the application has been adequate.						
20. The training documents that I can consult through the application are adapted to my needs.						
21. Do you consider that the application lacks any functionality? If "Yes", please tell us what functionality you would like us to include:						
USER EXPERIENCE WITH THE HEALTH APPLICATION						
22. The application provides me with all the tools I need to take care of my health.						
23. Since I use the application, I feel that I take better care of my health.						
24. The use of the application makes me feel accompanied by my health care team.						
25. Consulting my doubts through the application makes me feel more confident.						
26. The pending tasks that the application shows me make me feel overloaded.						
27. Receiving messages from my health care team through the application makes me feel overwhelmed.						
28. I would recommend the use of the app to other patients.						

Table S2. Checklist for Reporting Results of Internet E-Surveys (CHERRIES) [19].

Checklist Item	***Explanation***	***Page Number***
Describe survey design	Describe target population, sample frame. Is the sample a convenience sample? (In "open" surveys this is most likely.)	3, 5
IRB approval	Mention whether the study has been approved by an IRB.	3
Informed consent	Describe the informed consent process. Where were the participants told the length of time of the survey, which data were stored and where and for how long, who the investigator was, and the purpose of the study?	4, 11
Data protection	If any personal information was collected or stored, describe what mechanisms were used to protect unauthorized access.	11
Development and testing	State how the survey was developed, including whether the usability and technical functionality of the electronic questionnaire had been tested before fielding the questionnaire.	3
Open survey versus closed survey	An "open survey" is a survey open for each visitor of a site, while a closed survey is only open to a sample which the investigator knows (password-protected survey).	3

Contact mode	Indicate whether or not the initial contact with the potential participants was made on the Internet. (Investigators may also send out questionnaires by mail and allow for Web-based data entry.)	3-4
Advertising the survey	How/where was the survey announced or advertised? Some examples are offline media (newspapers), or online (mailing lists – If yes, which ones?) or banner ads (Where were these banner ads posted and what did they look like?). It is important to know the wording of the announcement as it will heavily influence who chooses to participate. Ideally the survey announcement should be published as an appendix.	-
Web/E-mail	State the type of e-survey (eg, one posted on a Web site, or one sent out through e-mail). If it is an e-mail survey, were the responses entered manually into a database, or was there an automatic method for capturing responses?	-
Context	Describe the Web site (for mailing list/newsgroup) in which the survey was posted. What is the Web site about, who is visiting it, what are visitors normally looking for? Discuss to what degree the content of the Web site could pre-select the sample or influence the results. For example, a survey about vaccination on a anti-immunization Web site will have different results from a Web survey conducted on a government Web site	-
Mandatory/voluntary	Was it a mandatory survey to be filled in by every visitor who wanted to enter the Web site, or was it a voluntary survey?	3
Incentives	Were any incentives offered (eg, monetary, prizes, or non-monetary incentives such as an offer to provide the survey results)?	-
Time/Date	In what timeframe were the data collected?	1, 3
Randomization of items or questionnaires	To prevent biases items can be randomized or alternated.	-
Adaptive questioning	Use adaptive questioning (certain items, or only conditionally displayed based on responses to other items) to reduce number and complexity of the questions.	-
Number of Items	What was the number of questionnaire items per page? The number of items is an important factor for the completion rate.	3; Sup. Table S1
Number of screens (pages)	Over how many pages was the questionnaire distributed? The number of items is an important factor for the completion rate.	2 pages: Sup. Table S1
Completeness check	It is technically possible to do consistency or completeness checks before the questionnaire is submitted. Was this done, and if "yes", how (usually JAVAScript)? An alternative is to check for completeness after the questionnaire has been submitted (and highlight mandatory items). If this has been done, it should be reported. All items should provide a non-response option such as "not applicable" or "rather not say", and selection of one response option should be enforced.	-

Review step	State whether respondents were able to review and change their answers (eg, through a Back button or a Review step which displays a summary of the responses and asks the respondents if they are correct).	-
Unique site visitor	If you provide view rates or participation rates, you need to define how you determined a unique visitor. There are different techniques available, based on IP addresses or cookies or both.	Login - password
View rate (Ratio of unique survey visitors/unique site visitors)	Requires counting unique visitors to the first page of the survey, divided by the number of unique site visitors (not page views!). It is not unusual to have view rates of less than 0.1 % if the survey is voluntary.	-
Participation rate (Ratio of unique visitors who agreed to participate/unique first survey page visitors)	Count the unique number of people who filled in the first survey page (or agreed to participate, for example by checking a checkbox), divided by visitors who visit the first page of the survey (or the informed consents page, if present). This can also be called "recruitment" rate.	-
Completion rate (Ratio of users who finished the survey/users who agreed to participate)	The number of people submitting the last questionnaire page, divided by the number of people who agreed to participate (or submitted the first survey page). This is only relevant if there is a separate "informed consent" page or if the survey goes over several pages. This is a measure for attrition. Note that "completion" can involve leaving questionnaire items blank. This is not a measure for how completely questionnaires were filled in. (If you need a measure for this, use the word "completeness rate".)	85/106
Cookies used	Indicate whether cookies were used to assign a unique user identifier to each client computer. If so, mention the page on which the cookie was set and read, and how long the cookie was valid. Were duplicate entries avoided by preventing users access to the survey twice; or were duplicate database entries having the same user ID eliminated before analysis? In the latter case, which entries were kept for analysis (eg, the first entry or the most recent)?	Yes
IP check	Indicate whether the IP address of the client computer was used to identify potential duplicate entries from the same user. If so, mention the period of time for which no two entries from the same IP address were allowed (eg, 24 hours). Were duplicate entries avoided by preventing users with the same IP address access to the survey twice; or were duplicate database entries having the same IP address within a given period of time eliminated before analysis? If the latter, which entries were kept for analysis (eg, the first entry or the most recent)?	Yes
Log file analysis	Indicate whether other techniques to analyze the log file for identification of multiple entries were used. If so, please describe.	-

Registration	In “closed” (non-open) surveys, users need to login first and it is easier to prevent duplicate entries from the same user. Describe how this was done. For example, was the survey never displayed a second time once the user had filled it in, or was the username stored together with the survey results and later eliminated? If the latter, which entries were kept for analysis (eg, the first entry or the most recent)?	Yes
Handling of incomplete questionnaires	Were only completed questionnaires analyzed? Were questionnaires which terminated early (where, for example, users did not go through all questionnaire pages) also analyzed?	Yes
Questionnaires submitted with an atypical timestamp	Some investigators may measure the time people needed to fill in a questionnaire and exclude questionnaires that were submitted too soon. Specify the timeframe that was used as a cut-off point, and describe how this point was determined.	No
Statistical correction	Indicate whether any methods such as weighting of items or propensity scores have been used to adjust for the non-representative sample; if so, please describe the methods.	No

Table S3. Summary model factor analysis.

	MR1	MR2	MR3	MR4
SS loadings	6.353	2.711	3.580	3.163
Proportion Variance	0.235	0.100	0.133	0.117
Cumulative Variance	0.235	0.585	0.368	0.485
Proportion Explained	0.402	0.171	0.226	0.200
Cumulative Proportion	0.402	1.000	0.628	0.829

Table S4. SUS score and overall and individual domain mean pSUAPP scores according to participants sociodemographic characteristics.

	**Age**				**Sex**			**Education**			
	**18-44**	**45-64**	**>65**	**p-value**	**Men**	**Women**	**p-value**	**Higher**	**Secondary**	**Primary**	**p-value**
SUS, mean (SD)	69.2 (14.1)	72.3 (13.2)	64.2 (12.7)	0.220	70.0 (15.0)	70.7 (12.2)	0.824	70.8 (15.0)	69.6 (10.8)	69.6 (10.4)	0.920
pSUAPP, mean (SD)	74.5 (12.4)	80.9 (11.7)	82.3 (11.1)	0.078	80.0 (13.7)	78.2 (10.7)	0.506	77.9 (13.1)	79.5 (9.89)	83.3 (10.2)	0.366
pSUAPP-reduced	73.9 (15.6)	79.7 (12.7)	81.7 (11.7)	0.172	79.1 (15.4)	77.2 (12.0)	0.557	77.2 (15.0)	79.0 (11.6)	80.0 (9.59)	0.765
Domains											
First contact	77.7 (13.3)	85.6 (14.1)	87.0 (12.6)	0.059	85.0 (15.0)	81.9 (13.4)	0.316	82.1 (15.3)	82.5 (12.0)	89.6 (10.6)	0.245

Registration	71.9 (15.5)	76.5 (15.7)	81.9 (17.5)	0.362	76.9 (16.1)	74.1 (15.4)	0.459	73.9 (15.8)	72.1 (15.2)	85.8 (12.5)	0.048
Features	80.2 (14.5)	87.4 (13.8)	87.5 (13.1)	0.134	85.1 (14.6)	85.2 (13.7)	0.973	84.1 (15.2)	88.1 (9.98)	85.9 (14.3)	0.607
Experience	69.6 (16.0)	75.0 (12.2)	76.2 (13.6)	0.236	73.6 (16.4)	73.3 (11.0)	0.933	73.0 (15.0)	76.4 (11.3)	69.9 (9.32)	0.406
